# Daily associations between salivary cortisol and electroencephalographic-assessed sleep: a 15-day intensive longitudinal study

**DOI:** 10.1093/sleep/zsae087

**Published:** 2024-04-08

**Authors:** Yang Yap, Natasha Yan Chi Tung, Lin Shen, Bei Bei, Andrew Phillips, Joshua F Wiley

**Affiliations:** School of Health and Biomedical Sciences, RMIT University, Melbourne, VIC, Australia; Inner Eastern Psychology, Melbourne, VIC, Australia; School of Psychological Sciences and Turner Institute for Brain and Mental Health, Monash University, Melbourne, VIC, Australia; School of Psychological Sciences and Turner Institute for Brain and Mental Health, Monash University, Melbourne, VIC, Australia; School of Psychological Sciences and Turner Institute for Brain and Mental Health, Monash University, Melbourne, VIC, Australia; School of Psychological Sciences and Turner Institute for Brain and Mental Health, Monash University, Melbourne, VIC, Australia

**Keywords:** sleep, home EEG, cortisol, daily design

## Abstract

**Study Objectives:**

Current evidence suggests that cortisol levels are bi-directionally associated with sleep. However, the daily, naturalistic cortisol-sleep associations remain unclear, as current evidence is mostly cross-sectional. This study tested whether pre-sleep cortisol predicts sleep duration and quality, and whether these sleep parameters predict the following day’s diurnal cortisol slope using a 15-day intensive longitudinal design with electroencephalographic measures and saliva sampling.

**Methods:**

Ninety-five young adults (*M*_age_ = 20.48 ± 1.59 years) provided saliva samples at awakening and pre-sleep over 14 consecutive days, providing 2345 samples (85% viable). The Z-Machine Insight + was used to record over 900 nights of total sleep time (TST) and sleep efficiency (SE). Multilevel models tested these data at the between- and within-person levels.

**Results:**

Higher pre-sleep cortisol predicted shorter TST (*p* < .001) and lower SE (*p* < .001) at the within-person level. Individuals with shorter average TST (*p = *.007) or lower average SE (*p < *.001) had flatter diurnal cortisol slopes, compared to those with longer average TST or higher average SE. Follow-up analyses showed that individuals with shorter average TST (vs. longer average TST) had higher pre-sleep cortisol levels (*p* = .01).

**Conclusions:**

Our findings provide evidence that pre-sleep cortisol is associated with sleep duration and quality at the within-individual level. Furthermore, individuals with short or poor sleep had flatter diurnal cortisol slopes. Although the effect sizes are small, these findings show the naturalistic associations between sleep and cortisol in a relatively healthy sample. These findings suggest that sleep maintains the regulation of the stress-response system, which is protective against mental and physical disorders.

## Graphical Abstract

**Graphical Abstract:**
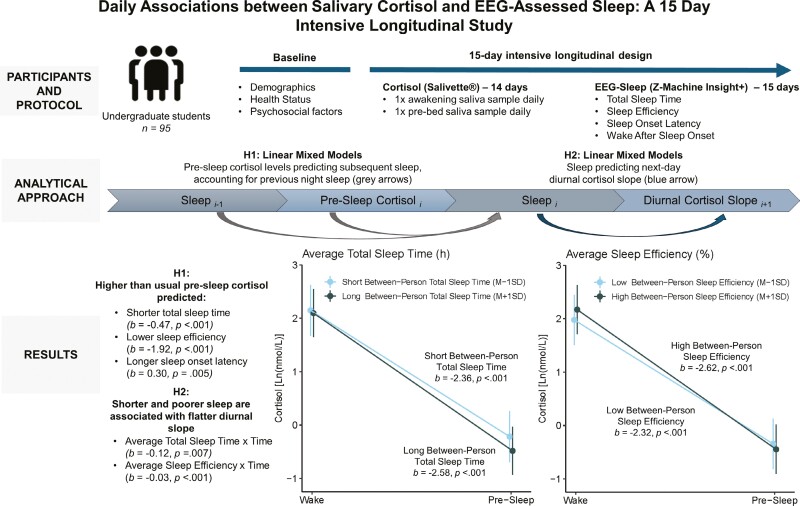

**Statement of Significance**

This study examined the daily associations between cortisol and sleep using a daily intensive longitudinal design with saliva sampling across 14 consecutive days and electroencephalographic sleep recordings across 15 consecutive nights. Compared to one’s own average, higher pre-sleep cortisol levels predicted subsequent shorter and poorer sleep quality that night. Individuals with shorter or poorer quality sleep on average had significantly flatter diurnal cortisol slope, which is indicative of a dysregulated stress-response system. The effect sizes are small, which could be attributable to the naturalistic design and the healthy sample. Nonetheless, these findings reinforce the importance of good sleep in regulating the stress-response system and maintaining optimal health.

The Hypothalamus-Pituitary-Adrenal (HPA) axis is the main stress-response system. Dysregulation in the HPA axis is associated with poor physical and mental health [1]. The HPA activity is often measured using cortisol, which is a primary end product of the HPA axis, given its responses to acute and chronic stressors [1, 2]. Current studies suggest a bi-directional association between cortisol and sleep, such that higher cortisol levels can impair sleep, and poor quality or short sleep can lead to dysregulated cortisol levels [3–5]. However, the associations between day-to-day variations in cortisol and sleep under naturalistic conditions are not well understood, given that most evidence is based on cross-sectional or laboratory designs. Most field studies of sleep have not used electroencephalographic (EEG) measures, and no studies to date have incorporated daily cortisol and EEG sleep measures across multiple days. Thus, this study examined whether pre-sleep cortisol levels predict subsequent EEG-assessed sleep, and whether EEG-assessed sleep predicts next-day diurnal cortisol, using a 15-day, intensive longitudinal design.

Higher daily stress is linked with shorter sleep and poorer sleep quality [6–9]. One study showed that self-reported pre-sleep somatic symptoms (e.g. sweating and heart racing) mediate this association [10]. One explanation for these findings is that the experience of stressors near bedtime activates the HPA axis, leading to increased cortisol levels and physiological arousal that disrupt sleep [11]. This interpretation is supported by several cross-sectional studies showing that higher evening or bedtime cortisol levels are associated with shorter and poorer quality sleep from self-report or actigraphy measures [12, 13]. Additionally, individuals with insomnia have higher evening and nocturnal cortisol compared to healthy controls [14, 15], and higher evening and nocturnal cortisol levels are associated with more awakenings at night [14]. Compared to healthy samples, individuals with Cushing Syndrome have higher actigraphy-assessed sleep fragmentation [16], longer PSG-assessed stage N2 sleep, and shorter PSG-assessed stage N3 and REM sleep [17]. After 12 months of treatment to reduce hypercortisolemia, these individuals had significant decrease in N2 sleep and longer N3 sleep [17]. Although these findings show the associations between increased cortisol levels and impaired or altered sleep, more research is needed to determine the temporal order, especially in naturalistic settings.

Cortisol secretion is endogenously driven and has a circadian rhythm; under constant routine, cortisol levels are high at awakening, peaking about 30–40 minutes post-awakening, and gradually decreasing across the day with the lowest levels around bedtime [1, 18]. Cortisol levels can be affected by sleep, with research showing that short or poor-quality sleep are associated with lower cortisol levels at awakening [5, 19–22], higher evening or bedtime cortisol levels [5, 23–25], slower daytime cortisol decline [22], and a flatter diurnal cortisol slope [5, 20, 21]. A flatter diurnal cortisol slope, which could be due to lower awakening cortisol levels, higher evening cortisol levels, or both, is indicative of dysregulated HPA activity and has been associated with poorer mental and physical health outcomes [1]. For example, experimental sleep restriction studies showed that sleep opportunities restricted to 3–4 hours significantly reduced morning cortisol levels, increased evening cortisol levels, and delayed the decline in daytime cortisol [22, 23]. These findings are supported by a recent review on the effects of sleep on 24-hour cortisol in men, showing that experimental sleep restrictions (i.e. from one night of total sleep deprivation to 4–14 days of 4–6 hours of sleep opportunities) can increase evening cortisol levels [26].

Experimentally restricting sleep opportunities in laboratory settings cannot be generalized to naturalistic settings, and only a few daily studies to date have tested the associations between sleep and cortisol. A 3-day study in young adults found that shorter average sleep duration was associated with a flatter diurnal cortisol slope, and that between- and within-person shorter sleep duration predicted lower next-day cortisol at awakening [21]. Another 3-day study had similar findings, where shorter between- and within-person actigraphic sleep duration predicted a flatter diurnal cortisol slope [20]. Although these findings show the daily associations between sleep and cortisol in naturalistic settings, they may not fully capture the variations in cortisol and sleep over longer periods. Emerging evidence shows that 10 days of cortisol sampling is needed to reliably detect both between- and within-person differences in diurnal cortisol [27], and a minimum of one week is needed to capture variation in sleep [28]. Furthermore, previous daily studies have relied on self-reported or actigraphic measures of sleep, which may not be as accurate as EEG measures. Thus, this study examined the cortisol-sleep associations with 14 days of saliva sampling and 15 nights of EEG-assessed sleep. Specific hypotheses were: (1) higher pre-sleep cortisol levels (at the between- and within-person levels) will predict same-night shorter total sleep time (TST) and lower sleep efficiency (SE) and (2) shorter TST and lower SE (at the between- and within-person levels) will predict a flatter next-day diurnal cortisol slope.

## Materials and Methods

### Participants

Ninety-five undergraduate students in Victoria, Australia, participated in the Stress and Health Study conducted between February 2019 and June 2020 [29]. This study focuses on stress, resilience, and health behaviors in emerging adults who recently relocated for tertiary education, and therefore the sample was predominantly international students. A priori power analyses with α = 0.05, 80% power as the target, and 10 predictors total, testing a single predictor with Cohen’s *f*^2^ effect sizes of 0.05 (small-to-medium, equivalent to a correlation of *r* = .*2*0, a “small” correlation) and 0.15 (the conventional cut off for a medium effect size) required 167 and 63 independent observations, respectively. Assuming a 75% completion rate on average over 14 days and intraclass correlation coefficients of .2 or .4, 75 participants would provide 271 and 164 effective independent observations, respectively, achieving 80% power. Additional participants were recruited to account for potential attrition, missing data, and aims not related to this paper. This study was not preregistered.

**Figure 1.**
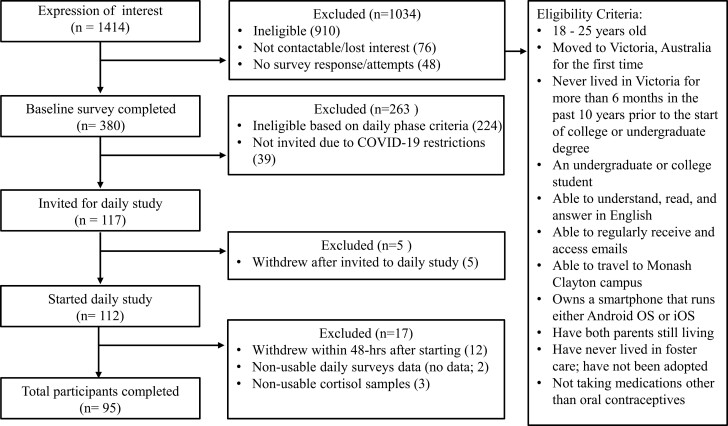
Summary of the Stress and Health Study recruitment flowchart.

Figure 1 summarizes the eligibility criteria and participant flowchart. Of the 910 participants excluded in the initial screening, 76% were due to no response to consent, duplicates, or did not provide contact details. The following three main reasons for exclusion were: not being an undergraduate or college student (7%), living in Victoria for more than 6 months in the past 10 years prior to the start of college or undergraduate degree (6%), and not within the age of 18–25 years (3%). Table 1 summarizes the participant characteristics.

**Table 1.** Sample Characteristics (*N* = 95)

Variables	*M* (SD)/*N* (%)	*n* (ICC)
Age (years)	20.48 (1.59)	95
Female (vs. male)	75 (78.9%)	95
International Student (vs. interstate)	88 (92.6%)	95
Race		95
Asian	81 (85.3%)	–
White	8 (8.4%)	–
Others	6 (6.3%)	–
Not working (vs. working)	75 (78.9%)	95
Never Smoked (vs. current/Former)	91 (95.8%)	95
Not using oral contraceptive (vs. using)	90 (94.7%)	95
Alcohol risk		95
Abstainer	22 (23.2%)	—
Moderate	62 (65.3%)	—
At-risk	11 (11.6%)	—
Body mass index (kg/m^2^)	21.84 (3.43)	95
Depression (possible range: T-Score 31.7–81.3)	52.75 (9.24)	95
Anxiety (possible range: T-Score 31.7–81.3)	56.07 (9.09)	95
Average daily stress (possible range: 0–10)	2.14 (1.49)	95
Average daily negative affect (possible range: 1–5)	1.48 (0.51)	95
Total sleep time (h)	6.21 (0.90)	943 (.29)
Sleep onset latency (min)	22.48 (11.62)	1100 (.21)
Wake after sleep onset (min)	48.36 (22.32)	943 (.32)
Sleep efficiency (%)	83.90 (5.76)	943 (.33)
Awakening cortisol (nmol/L)	11.32 (14.68)	1142 (.23)
Pre-sleep cortisol (nmol/L)	1.29 (1.93)	1203 (.34)

ICC, intraclass correlations; *n,* number of observations. Values in parenthesis for *n*(ICC) indicate the percent of variance within individuals. Sleep Onset Latency, Wake After Sleep Onset, Sleep Efficiency, and Cortisol presented are raw values.

### Design and procedure

The study procedures were approved by Monash University Human Research Ethics Committee, and all participants provided informed consent. This study used a daily intensive longitudinal design. First, participants completed a baseline survey consisting of questions related to sociodemographic details, psychosocial factors, and health status. Eligible participants were then invited to the daily phase of the study, commencing on a Monday and ending 15 days after (i.e. Tuesday). On the first day, participants attended a 1-hour orientation to the study, where they were shown how to provide saliva samples and wear the EEG sleep monitoring device (Z-Machine Insight+). For saliva cortisol collection, participants were instructed to provide saliva samples twice (i.e. at awakening and at pre-sleep) per day for 14 consecutive days. Once collected, participants wrote the date and time on the Salivette tubes, took photographs of those tubes, and uploaded them onto the MetricWire app to allow for digital timestamp and cross-examination of the written collection date and time. Participants were instructed not to (1) provide the samples when waking more than ± 2 hours outside of habitual wake time or sleeping more than ± 4 hours outside of habitual bedtime; (2) brush their teeth, eat, or drink within the 30 minutes prior to sampling; and (3) have any dental work done within the 24 hours prior to sampling. Participants were instructed not to consume any major meals, alcohol, nicotine (smoking), caffeine, and/or medication within the 60 minutes before saliva collection, and not to chew gum, perform vigorous physical activity, or drink water right before saliva collection. Participants were instructed to report any of these violations in the pre-sleep survey via MetricWire each day. For the EEG sleep monitoring device, participants were asked to wear the machine for 15 consecutive nights. Participants were instructed to clean their skin with the provided alcohol wipes and apply the sensors approximately 30 minutes before bedtime. Participants were instructed only to start the recording when they attempted to sleep.

### Measures

#### Salivary cortisol.

Salivary samples were collected using Salivettes [30] twice daily for 14 days: (1) at awakening within ± 2 hours of habitual wake time, and (2) at bedtime within ± 4 hours of habitual sleep time. The time window for saliva collection was based on a previous study to partially control for the influence of circadian phase on cortisol [31]. The decision to collect two samples per day was based on a previous study demonstrating that two cortisol samples taken per day for 10 days can better detect between- and within-person differences in diurnal cortisol slope compared to taking five samples per day for 4 days [27]. Diurnal slopes measured based on two cortisol samples taken at wake time and 21:00 correlated .97 and .99 with diurnal slopes measured using four and three samples, respectively. Thus, this approach maximizes the power to detect both between- and within-person differences while minimizing the cost of the collection and assay analysis, as well as participant impact. Samples were removed and not sent for assay analysis if (1) they were collected outside of the respective habitual wake or sleep time, (2) > 1-hour discrepancy between the digital timestamp and written label, and (3) > 30 minutes after waking. In total, 85% of the collected saliva samples were usable for analyses.

#### Sleep.

EEG-estimated TST and SE (i.e. $\frac{TST}{Time\;\;\;in\;\;\;Bed}\;\times 100$) were measured using the Z-Machine Insight+. Raw EEG signals were automatically scored as sleep or wake through an automated sleep–wake detection algorithm (Z-ALG) [32]. Previous studies showed that the Z-ALG has high sensitivity (95.5%) and specificity for determining sleep (92.5%). High agreement between Z-ALG and polysomnography technologists is observed, with *r = *.95 for TST and *r = *.93 for SE [32]. On nights where we identified sensor errors or battery issues, TST, wake after sleep onset (WASO), and SE for that night were set as missing. Prior to removing participants with missing saliva samples, 74% of EEG recordings for TST, WASO, and SE, and 86% of sleep onset latency (SOL), were usable for analyses.

#### Covariates.

Covariates were determined based on previous studies showing their associations with cortisol and sleep: age (years) [33, 34], sex (coded as male/female) [33, 34], race/ethnicity (coded as Asian/White/Others) [35, 36], alcohol risk (coded as abstainer/moderate/at-risk) [33, 37], smoking (coded as never vs. current/former) [33, 38], time spent in Victoria, subjective socioeconomic status [36], COVID-19 period (coded as pre [before Victoria lockdown 2020 March 8] vs. during) [39], work status (coded as working/not working) [36], student status (coded as international/interstate) [40], oral contraceptive use (coded as using vs. not using) [33], body mass index (kg/m^2^) [38, 41], average daily stress [1, 6], average daily negative affect [42, 43], depressive symptoms [44, 45], anxiety symptoms [44, 45], and day of week [46]. Given that research has shown the associations between social jetlag (≥2 hours) and higher cortisol levels [47], participants’ daily circadian misalignment was accounted for in the models using the Composite Phase Deviation, which is a metric that combines both sleep irregularity and mistiming [48, 49]. Saliva collection adherence, such as consuming any major meals, alcohol, nicotine (smoking), caffeine, and/or medication 60 minutes before saliva collection, and chewing gum, engaging in vigorous physical activity, or drinking water right before saliva collection, were included (coded as no violations/violations) [33, 42].

### Analytical approach

All analyses were conducted in R v4.2.0, and linear mixed models (using lme4 v1.1–29) were used to analyze these data. Degrees of freedom and significance testing were performed using lmerTest v3.1-3. Pre-sleep cortisol and sleep variables were separated into between-person (i.e. the individual’s average cortisol or sleep values across the study period) and within-person levels (i.e. the deviation of the cortisol or sleep values from the individual’s average). Given the skewness, cortisol was log-transformed, and SE was winsorized (top and bottom 0.5%).

For models testing sleep as the outcome (i.e. TST and SE), pre-sleep cortisol, the lagged sleep outcome (i.e. accounting for the previous night sleep variable [*sleep i*-1] to strengthen the test of directionality), and covariates were entered as predictors of sleep that night. Model comparison through stepwise addition of sleep lags (one to three lags) and Bayesian Information Criterion values showed that the model with the first-night lag was the most appropriate model. To test diurnal cortisol slope as the outcome, the models included the interaction between time (i.e. awakening and pre-sleep) and sleep (between- and within-person levels at the prior night [*sleep i*-1]), and covariates as predictors. Significant interaction effects were probed using simple slopes by testing longer than the sample average TST or higher than sample average SE (i.e. *M* + 1SD) and shorter than sample TST or lower than sample average SE (i.e. *M*-1SD). Mean differences between long/short TST or high/low SE within timepoints also were conducted to determine whether slopes were due to changes in awakening or pre-sleep cortisol levels (using emmeans v1.5.4). These analyses are repeated using conventional cutoffs using 6 and 8 hours. Follow-up analyses were planned for SOL and WASO if results for SE were significant. Due to skewness, SOL and WASO were square-root transformed and winsorized (top and bottom 0.5%). Cohen’s *f*^2^ effect size was calculated for all predictors. All adjusted models included between- and within-person predictors and covariates as fixed effects, whereas intercepts and within-person predictors were included as random effects. Convergence issues were resolved using the Nelder-Mead algorithm and by tightening tolerance values. Singularity issues were resolved by dropping the random effect variables with the lowest variance. Unadjusted models are reported in Supplementary S1.

## Results

Table 1 summarizes the participant characteristics. Participants were mostly females (72.0%), international students (92.6%), and of Asian descent (85.3%). Participants were generally healthy with most indicating they never smoked (95.8%), were moderate drinkers (65.3%), and were within the healthy BMI range (67.4% within normal range, 16.8% underweight, and 14.7% overweight). Most participants (94.7%) were not using oral contraceptives. Participants’ depression and anxiety symptoms were within normal levels (PROMIS T-Scores with a population mean = 50, *SD* = 10) [50]. Daily average stress levels and negative affect were similar to young adults and working adults reported in other daily studies [6, 9, 51]. On average, participants’ TST was 6.23 hours, and SE was 83.9%. The participants’ average TST is below the recommended sleep hours for young adults (i.e. between 7 and 9 hours) [52], which could be due to juggling competing demands such as study, social activities, and employment as university students. The short average TST could also be due to differences in the measurements used; for example, our previous work showed that the average self-reported TST was 7.44 ± 0.96 hours, whereas EEG-assessed sleep was 6.23 ± 0.90 hours [29]. These differences are likely due to the overestimation of self-reported sleep measures [53].

### Pre-sleep Cortisol Predicting Sleep

Table 2 (first four rows) summarizes the adjusted models of pre-sleep cortisol (between- and within-person levels) predicting sleep. Within-person effects showed that a higher pre-sleep cortisol level was significantly associated with shorter subsequent TST (*p* < .001) and lower SE (*p* < .001). This indicates that on days when participants had higher than usual pre-sleep cortisol levels, they had shorter sleep duration and poorer SE that night. Given the significant association with SE, follow-up analyses were conducted on SOL and WASO. Higher than usual pre-sleep cortisol predicted longer SOL (*p* = .005) but not WASO. No significant results emerged at the between-person level. Results for unadjusted models (Supplementary Table S1) showed that higher pre-sleep cortisol predicted shorter TST and lower SE. However, unadjusted results for SOL were nonsignificant. Results for covariates are reported in Supplementary Table S2.

**Table 2.** Multilevel Model Testing Cortisol as Predictor and Outcome of Sleep (N = 95)

	Between-person level	Within-person level
	*Pre-sleep cortisol as predictor of sleep*	
TST (h)	−0.47 [−0.95, 0.02] *p = *.06, *f*^2^ = .02	−0.39 [−0.55, −0.24] *p < .*001*, f*^*2*^* = *0.03
SE (%)	−1.86 [−4.99, 1.28] *p = *.25, *f*^2^ = .01	−1.92 [−2.75, −1.09] *p < .*001*, f*^*2*^* = *0.02
SOL ($\sqrt{min}$)^†^	0.11 [−0.57, 0.78] *p = *.75, *f*^2^ < .01	0.30 [0.09, 0.52] *p = .*005*, f*^*2*^* = *0.02
WASO ($\sqrt{min}$)^†^	0.22 [−0.61, 1.06] *p = *.60, *f*^2^ < .01	0.14 [−0.08, 0.36] *p = *.14, *f*^2^ < .01
	*Sleep × Time as Predictor of Diurnal Cortisol Slope*	
TST(h) × time	−0.12 [−0.21, −0.03] *p = .*007*, f*^*2*^* = *0.01	0.02 [−0.04, 0.08] *p = *.52, *f*^2^ < .01
SE (%) × time	−0.03 [−0.04, −0.01] *p < .*001*, f*^*2*^*=*0.01	−0.01 [−0.02, 0.01] *p = *.26, *f*^2^ < .01
SOL($\sqrt{min}$) × time^†^	0.01 [−0.06, 0.08] *p = *.83, *f*^2^ < .01	0.02[−0.02, 0.07] *p = *.31, *f*^2^ < .01
WASO($\sqrt{min}$) × time^†^	0.08 [0.02, 0.13] *p = .005, f*^*2*^* = *0.01	0.02 [−0.02, 0.07] *p = *.34, *f*^2^ < 0.01

Results are reported as unstandardized coefficients, [95% Confidence Interval], *p*-values, *f*^2^ effect size. Values in italics denote significant results. Cortisol values are log-transformed. SOL and WASO are square-root transformed. TST, total sleep time; SE, sleep efficiency; SOL, sleep onset latency; WASO, wake after sleep onset.

^†^Follow-up analyses gave significant results for sleep efficiency.

### Sleep Predicting Diurnal Cortisol Slope

**Figure 2.**
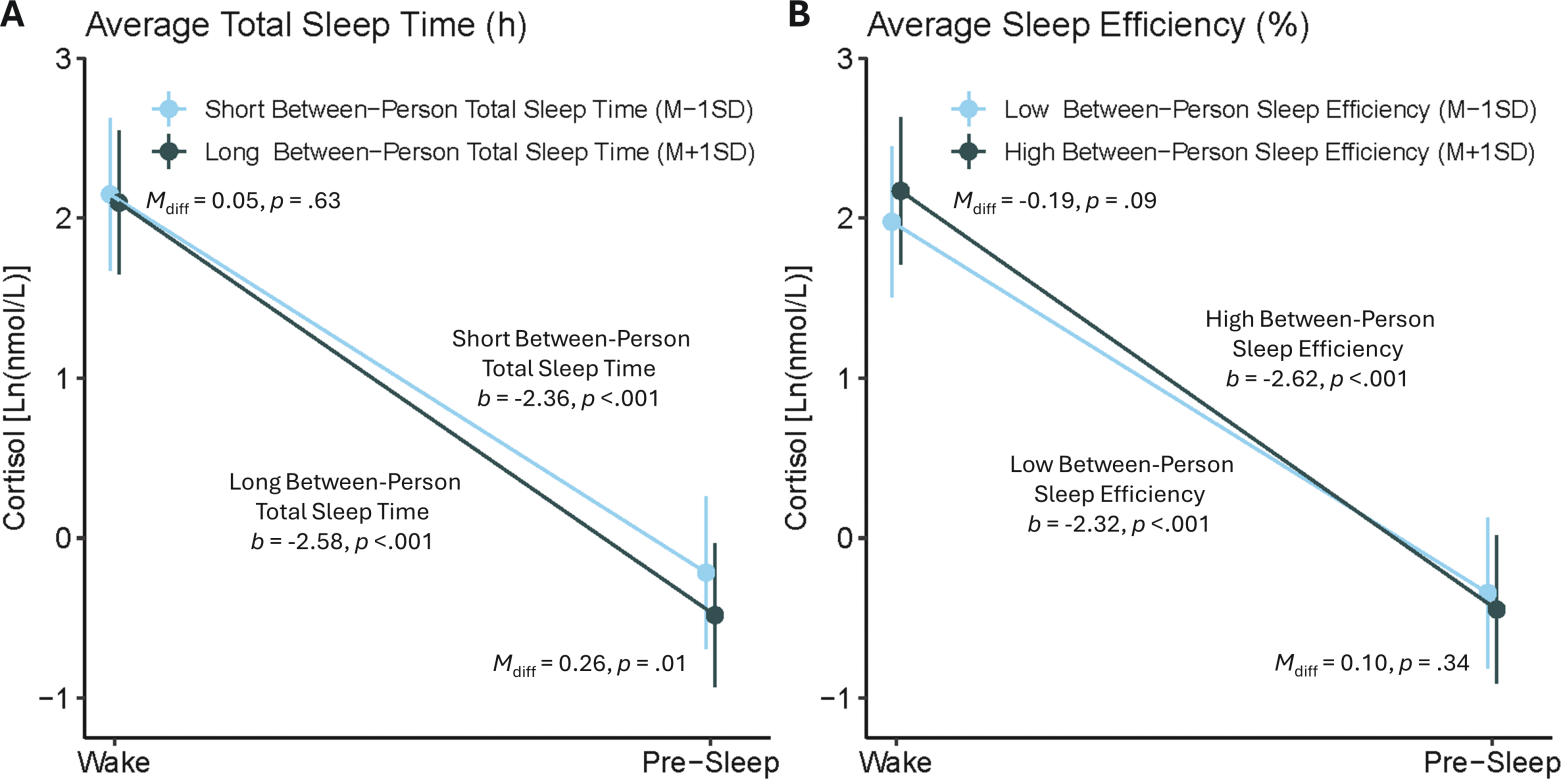
Panel A shows the interaction of between-person total sleep time and time (wake and pre-sleep) on cortisol. Panel B shows the interaction of between-person sleep efficiency and time (wake and pre-sleep) on cortisol.

Table 2 (last four rows) summarizes the adjusted models of the interaction between sleep (between- and within-person levels) and time (awakening and pre-sleep), predicting the next-day diurnal cortisol slope. A significant time × TST interaction effect on next-day cortisol (*p* = .007) emerged at the between-person level (Figure 2, Panel A). Specifically, individuals with shorter average TST had a flatter diurnal cortisol slope (*b = *−2.36, *p* < .001) compared to individuals with longer average TST (*b* = −2.58, *p* < .001). These findings are similar when standard cutoff was used for short average TST at 6 hours (*b* = −2.44, *p* < .001) and long average TST at 8 hours (*b* = −2.68, *p* < .001). A similar interaction emerged for SE at the between-person level (*p* < .001; Figure 2, Panel B). Individuals with a lower average SE had a significantly flatter diurnal cortisol slope (*b* = −2.32, *p* < .001) compared to individuals with higher average SE (*b* = −2.62, *p* < .001). Follow-up analyses were conducted to clarify whether the flatter slopes were due to lower cortisol levels upon awakening and/or higher cortisol levels during pre-sleep. Individuals with shorter average TST had significantly higher pre-sleep cortisol levels compared to individuals with longer average TST (*M*_diff_ = 0.26, *p* = .01); no significant differences emerged for awakening cortisol levels between long versus short TST. These findings are also similar when standard cutoff hours were used, with no significant difference at awakening (*M*_diff_ = 0.06, *p* = .46), whereas individuals who sleep 6 hours on average had higher cortisol levels during pre-sleep compared to individuals who sleep 8 hours on average (*M*_diff_ = 0.30, *p* = .01). For SE, although the slopes were significantly different from one another, the awakening (*M*_diff_ = −0.19, *p* = .09) and pre-sleep cortisol levels (*M*_diff_ = −0.10, *p* = .35) did not significantly differ between individuals with lower vs higher average SE.

Follow-up analyses were conducted on SOL and WASO given the significant effects of time × SE on diurnal cortisol. Results showed a significant time × WASO interaction at the between-person level (*p* = .005). Specifically, longer average WASO (between-person effects; *b* = −2.36, *p* < .001) is associated with a flatter diurnal cortisol slope compared to shorter average WASO (*b =* −2.58, *p < *.001). Longer average WASO (vs. shorter average WASO; *M*_diff_ = 0.23, *p* = .03) is associated with lower awakening cortisol, but not pre-sleep cortisol levels. No significant findings emerged for SOL.

Results for unadjusted models showed a significant interaction of between-person TST, SE, or WASO and Time on diurnal cortisol, and the directions were similar to the adjusted models (Supplementary Table S1). Results for covariates are reported in Supplementary Table S3.

## Discussion

This study tested the daily cortisol-sleep associations using an intensive longitudinal design with 14 days of cortisol sampling and 15 nights of EEG-assessed sleep. We found that higher pre-sleep cortisol levels predicted subsequent shorter TST, lower SE, and longer SOL at the within-person level. No significant results emerged for average pre-sleep cortisol levels predicting sleep (i.e. between-person effects). Individuals with shorter TST and lower SE on average had a flatter diurnal cortisol slope across the day (i.e. between-person effects). Follow-up analyses showed that longer average WASO was associated with flatter diurnal slope. No significant results emerged for nightly variations in TST or SE predicting diurnal cortisol slope.

The current findings revealed the temporal relationships between cortisol and sleep at a daily level, extending from previous cross-sectional studies [12–14]. Specifically, regardless of individuals’ average pre-sleep cortisol levels, on nights where individuals had higher than usual pre-sleep cortisol levels, they had shorter EEG-estimated sleep duration and lower SE that night. The directionality of these findings was further strengthened by the inclusion of lagged outcomes, meaning that the effects on sleep observed that night were independent of the previous night’s sleep. These associations may be due to a direct effect of cortisol on sleep. Cortisol secretion is associated with physiological arousals (e.g. increased heart rate; temperature) and a subsequent increase in feelings of alertness or activeness [54]. These physiological and cognitive arousals can disrupt sleep. This interpretation is supported by our follow-up analyses showing that higher pre-sleep cortisol levels predicted longer time taken to fall asleep. Together, these findings showed that higher-than-usual cortisol levels around bedtime predict poorer and shorter subsequent sleep.

Individuals with short or poor sleep on average had flatter diurnal cortisol slopes across the day compared to individuals with long or good sleep on average. These findings are in line with previous studies showing shorter average sleep duration or self-reported poorer sleep quality associated with flatter diurnal slope [5, 19–23]. However, we did not find any significant within-person effects, which is contrary to previous findings showing shorter than usual sleep duration predicted flatter diurnal slope the following day [20]. Inconsistencies in these findings could be due to the differences in cortisol saliva sampling frequency (14 days in this study vs. 3 days in the previous study) and sleep measurements (EEG vs. actigraphy). It is possible that the participants did not experience large nightly fluctuations in sleep during this period (i.e. TST *i*SD = 1.12), which could significantly impact the next-day cortisol slope. Nonetheless, the associations between average sleep and diurnal cortisol slope are observed (i.e. between-person effects), suggesting the long-term impact of sleep on the HPA axis. For example, an individual who sleeps 1h shorter than their average sleep duration on a given night may not have a significant impact on their HPA axis regulation the following day; however, over a longer period, individuals with shorter average sleep duration (e.g. 5 hours) may have a dysregulated HPA axis compared to those with longer average sleep duration (e.g. 7 hours). More research is needed to confirm these explanations. Our follow-up analyses indicated that individuals with shorter average sleep duration (vs. longer average sleep duration) had significantly higher pre-bedtime cortisol, resulting in a flatter diurnal slope. Previous studies have shown that individuals with partial or total sleep restriction have slower decline of cortisol concentrations throughout the day, resulting in higher evening cortisol levels, which reflect dysregulation of the negative feedback regulation of the HPA axis [22–25]. Previous studies have found that nighttime awakenings are associated with a subsequent increase in cortisol levels, followed by a temporary inhibition of cortisol secretion [3, 55]. Thus, it is possible that lower awakening cortisol levels could be due to the temporary inhibition of secretion after elevation of nighttime cortisol from the nighttime awakenings, as hypothesized by Backhaus and colleagues [19]. Supporting this explanation, our follow-up analyses indicated that individuals with high average WASO had significantly lower awakening cortisol levels the following day. Nonetheless, this hypothesis cannot be directly tested in our study, since cortisol was not sampled overnight, thus requiring further research. Collectively, these findings suggest that poor and short sleep are associated with a dysregulated HPA axis, as indicated by a dysregulated diurnal cortisol slope.

A key strength of our study was the collection of salivary cortisol across 14 consecutive days and the use of EEG sleep recordings across 15 consecutive nights, extending previous cross-sectional and daily studies. Specifically, this study was longer than most previous studies examining the cortisol-sleep relationship and used more accurate sleep measures compared to studies using self-report or actigraphic measures [5, 20, 21]. Together, these methods allowed a more reliable detection of within- and between-person differences in diurnal cortisol slope [27]. Our focus on maximizing adherence (85% usable cortisol data) and minimizing participant impact in cortisol collection also strengthened results [56]. Nonetheless, our study only examined cortisol levels twice daily, which cannot provide information on cortisol awakening responses or reliable estimates of total daily cortisol output. These results also cannot provide a full understanding of the HPA axis and other stress-response systems (e.g. the autonomic nervous system), as other physiological indicators were not examined. Although multiple covariates related to cortisol and sleep were adjusted for in our analyses, these findings may still be impacted by unexplored confounds, such as menstrual cycle phase, and should not be interpreted as causal [33]. For example, a meta-analysis showed that females in the follicular phase had higher circulating or tonic cortisol levels compared to females in the luteal phase [57], whereas females in the luteal phase showed significantly higher cortisol reactivity to psychosocial stressors compared to females in the follicular phase [33], Lastly, these findings cannot be generalized to clinical populations, individuals on medications, or to older populations that have different cortisol and sleep profiles [33, 58]. Future research is needed to account for these limitations to strengthen these findings by including additional physiological indicators, controlling for menstrual cycle phases, and examining different populations.

In summary, our findings provide a deeper understanding of the cortisol-sleep relationship in a naturalistic setting. Our results provide within-person evidence of higher cortisol levels at pre-sleep predicting shorter sleep, poorer sleep quality, and longer time to fall asleep that night. These findings support cortisol levels during pre-sleep as a potential mechanism for the association between daily stress and sleep. Moreover, individuals with shorter or poorer average sleep had a significantly flatter diurnal cortisol slope. It is worthwhile highlighting that the effect sizes are small, which could be attributable to the naturalistic design of the study and the relatively healthy sample. Nonetheless, these findings could inform future interventional research to verify these findings, such as daily interventions aiming to reduce cortisol levels to improve sleep. For example, meta-analyses suggest that mindfulness-based interventions can reduce cortisol levels [59, 60] and improve sleep duration and quality [61, 62]. It is possible that these interventions may improve sleep partly through reducing cortisol levels, although future studies are needed to test these mediating pathways. These findings also reinforce the importance and benefits of good sleep (both duration and quality) on HPA axis functioning. Improving sleep duration and quality may help maintain or improve the HPA axis regulation, thus lowering the risks of developing mental and physical disorders.

## Supplementary material

Supplementary material is available at *SLEEP* online.

zsae087_suppl_Supplementary_Tables

## Data Availability

Research materials are available at OSF https://doi.org/10.17605/OSF.IO/TZ48Y. The data underlying this article will be shared on reasonable request to the corresponding author.
