# Supplementary material for: Daily associations between salivary cortisol and electroencephalographic-assessed sleep: a 15-day intensive longitudinal study
Source: Sleep. 2024 Apr 8;47(9):zsae087. doi: 10.1093/sleep/zsae087 (PMC11381568; doi:10.1093/sleep/zsae087)
Supplement: zsae087_suppl_Supplementary_Tables [file zsae087_suppl_supplementary_tables.docx]

# Daily Associations between Salivary Cortisol and EEG-Assessed Sleep: A 15-Day Intensive Longitudinal Study

Dr Yang Yap^1^

Dr Natasha Yan Chi Tung^2^

Dr Lin Shen^3^

A/Prof Bei Bei^3^

A/Prof Andrew Phillips^3^

Dr Joshua F. Wiley^3^

^1^School of Health and Biomedical Sciences, RMIT University, Melbourne, Australia

^2^Inner Eastern Psychology, Melbourne, Australia

^3^School of Psychological Sciences and Turner Institute for Brain and Mental Health, Monash University, Melbourne, Australia

***Corresponding Author:***

*Dr Yang Yap
RMIT University
School of Health and Biomedical Sciences
Building 12, Level 7, Room 16B
124 La Trobe Street, Melbourne, 3000, VIC, Australia
yang.yap@rmit.edu.au*

Supplementary S1

| Table S1. *Multilevel Model Testing Cortisol as Predictor and Outcome of Sleep (N = 95)* | | |
| --- | --- | --- |
|  | **Between-Person level** | **Within-Person level** |
|  | *Pre-Sleep Cortisol as Predictor of Sleep* | |
| **TST (h)** | -0.26 [-0.63, 0.10]  *p =* .16, *f^2^*=.01 | **-0.39 [-0.53, -0.25]**  ***p* <.001, *f^2^=*0.03** |
|  |  |  |
| **SE (%)** | -0.47 [-2.87, 1.94]  *p =* .70, *f^2^*<.01 | **-1.84 [-2.62, -1.06]  *p <*.001, *f^2^=*0.02** |
|  |  |  |
| **SOL (**$\sqrt{\text{min}}$**)**^†^ | -0.02 [-0.46, 0.42]  *p =* .92, *f^2^*<.01 | 0.16 [-0.01, 0.33]  *p =* .08, *f^2^=*0.02 |
|  |  |  |
| **WASO (**$\sqrt{\text{min}}$**)**^†^ | -0.12 [-0.76, 0.52]  *p =* .71, *f^2^* <.01 | 0.13 [-0.07, 0.33]  *p =* .20, *f^2^* <.01 |
|  |  |  |
|  | *Sleep x Time as Predictor of Diurnal Cortisol Slope* | |
| **TST(h) x Time** | **-0.15 [-0.23, -0.08]**  ***p* <.001, *f^2^* *=*0.01** | 0.02 [-0.04, 0.07]  *p =* .54, *f^2^*<.01 |
|  |  |  |
| **SE (%) x Time** | **-0.03 [-0.04, -0.02]**  ***p <*.001, *f^2^=*0.01** | 0.004 [-0.01, 0.01]  *p =* .43, *f^2^*<.01 |
|  |  |  |
| **SOL(**$\sqrt{\text{min}}$**) x Time**^†^ | 0.02 [-0.04, 0.07]  *p =* .57, *f^2^*<.01 | -0.005 [-0.04, 0.03]  *p =* .81, *f^2^*<.01 |
|  |  |  |
| **WASO(**$\sqrt{\text{min}}$**) x Time**^†^ | **0.07 [0.03,0.11]**  ***p =* .001, *f^2^=*.01** | 0.02 [-0.01, 0.06]  *p =* .20, *f^2^*<.01 |
| *Note*. Results are from unadjusted models. Results are reported as unstandardized coefficients, [95% Confidence Interval], *p*-values, *f*^2^ effect size. Values in bold denote significant results. Cortisol values are log transformed. SOL and WASO are square-root transformed. TST = Total Sleep Time. SE = Sleep Efficiency. SOL = Sleep Onset Latency. WASO = Wake After Sleep Onset. ^†^ = Follow up analyses given significant results for Sleep Efficiency | | |

Supplementary S2

Table S2. Covariates as Predictors of EEG-assessed Sleep

|  | **TST** | **SE** | **SOL** | **WASO** |
| --- | --- | --- | --- | --- |
| Anxiety | -0.02 | -0.13 | 0.03 | 0.02 |
| Alcohol Risk (Ref: Abstinence) |  |  |  |  |
| At Risk | -0.17 | 1.72 | -0.67 | -0.33 |
| Moderate | -0.48 | 0.84 | -0.36 | -0.33 |
| Age | -0.05 | -0.24 | -0.07 | 0.11 |
| BMI | -0.01 | 0.00 | 0.01 | 0.03 |
| Between CPD | -0.11 | -1.93 | 0.01 | 0.51 |
| Within CPD | -0.23*** | -0.94** | -0.09 | 0.10 |
| Negative Affect | 0.73 | -0.41 | 0.48 | 0.28 |
| Stress | -0.20 | -0.40 | -0.02 | 0.05 |
| Student Status | -0.13 | -3.50 | 0.26 | 0.72 |
| Cort_Check | -0.44 | 0.81 | 0.46 | -0.71 |
| Covid | 0.06 | -0.33 | -0.05 | 0.12 |
| Employment | 0.25 | -0.84 | 0.48 | 0.48 |
| Depression | 0.02 | 0.21 | -0.02 | -0.04 |
| Smoking Status | 0.11 | 2.19 | 0.42 | -1.02 |
| Day of Week (Ref Monday) |  |  |  |  |
| Friday | 0.54** | 3.57 | -0.68 | -0.21 |
| Saturday | 0.72*** | 2.28 | -0.49 | 0.36 |
| Sunday | 0.43** | 1.66 | -0.53 | 0.41 |
| Thursday | 0.41* | 2.61 | -0.41 | -0.07 |
| Tuesday | 0.36* | 3.15 | -0.72 | -0.17 |
| Wednesday | 0.25 | 2.58 | -0.65 | -0.11 |
| Meds | 0.40 | 4.96 | -0.80 | -1.43 |
| Race (Ref: Asian) |  |  |  |  |
| Other | -0.14 | -3.27 | 0.36 | 0.69 |
| White | 0.68 | 2.80 | -0.30 | -0.24 |
| SES | 0.12 | 0.49 | -0.14 | 0.08 |
| Sex | -0.04 | -2.39 | 0.13 | 0.68 |
| Time in Melbourne | -0.02 | -0.38 | 0.11 | -0.12 |
| Lag Sleep | -0.13** | -0.14*** | -0.08 | -0.04 |

*Note*. Results are from unadjusted models. Results are reported as unstandardized coefficients. SOL and WASO are square-root transformed. TST = Total Sleep Time. SE = Sleep Efficiency. SOL = Sleep Onset Latency. WASO = Wake After Sleep Onset. CPD = Composite Phase Deviation. * = *p* <.05, ** = *p* <.01, *** = *p* <.001

Supplementary S3

Table S3. Covariates as Predictors of Next Day Cortisol Slope

|  | **TST** | **SE** | **SOL** | **WASO** |
| --- | --- | --- | --- | --- |
| Anxiety | 0.01 | 0.00 | 0.01 | 0.01 |
| Alcohol Risk (Ref: Abstinence) |  |  |  |  |
| At Risk | -0.03 | 0.01 | -0.04 | -0.01 |
| Moderate | 0.08 | 0.13 | 0.08 | 0.10 |
| Age | -0.06 | -0.06* | -0.05 | -0.05 |
| BMI | 0.00 | 0.00 | 0.00 | 0.00 |
| Between CPD | 0.09 | 0.07 | 0.12 | 0.12 |
| Within CPD Lag | -0.01 | -0.01 | -0.01 | -0.01 |
| Negative Affect | 0.03 | 0.00 | 0.02 | 0.02 |
| Stress | -0.01 | 0.00 | 0.00 | 0.00 |
| Student Status | -0.28 | -0.30 | -0.26 | -0.24 |
| Cort_Check | 0.01 | 0.02 | 0.01 | 0.03 |
| Covid | -0.11 | -0.10 | -0.10 | -0.09 |
| Employment | -0.04 | -0.06 | -0.06 | -0.08 |
| Depression | 0.00 | -0.01 | -0.01 | -0.01 |
| Smoking Status | 0.08 | 0.08 | 0.12 | 0.07 |
| Day of Week (Ref Monday) |  |  |  |  |
| Friday | 0.11 | 0.11 | 0.12 | 0.10 |
| Saturday | 0.02 | 0.03 | 0.04 | 0.02 |
| Sunday | -0.04 | -0.02 | -0.02 | -0.03 |
| Thursday | 0.01 | -0.01 | 0.01 | -0.01 |
| Tuesday | 0.06 | 0.05 | 0.05 | 0.03 |
| Wednesday | 0.04 | 0.04 | 0.05 | 0.03 |
| Meds | -0.09 | -0.08 | -0.16 | -0.17 |
| Race (Ref: Asian) |  |  |  |  |
| Other | -0.32 | -0.43* | -0.32 | -0.30 |
| White | 0.50* | 0.44* | 0.45* | 0.45* |
| SES | 0.05 | 0.04 | 0.03 | 0.03 |
| Sex | -0.05 | -0.07 | -0.02 | 0.01 |
| Time in Melbourne | -0.01 | -0.01 | -0.01 | -0.02 |

*Note*. Results are from unadjusted models. Results are reported as unstandardized coefficients. SOL and WASO are square-root transformed. TST = Total Sleep Time. SE = Sleep Efficiency. SOL = Sleep Onset Latency. WASO = Wake After Sleep Onset. CPD = Composite Phase Deviation. Each sleep variable represents the model in which the sleep x time variable was examined. * = *p* <.05
